# Case report: systemic tuberculosis caused by *Mycobacterium bovis* in a cat

**DOI:** 10.1186/s12917-018-1759-7

**Published:** 2019-01-05

**Authors:** Yesari Eroksuz, Ersoy Baydar, Baris Otlu, Murat Dabak, Hatice Eroksuz, Burak Karabulut, Canan Akdeniz Incili, Mehmet Ozkan Timurkan

**Affiliations:** 1 Department of Pathology, Faculty of Veterinary Medicine, Firat University, 23 200 Elazig, Turkey; 2 Department of Internal Medicine, Faculty of Veterinary Medicine, Balikesir University, Balikesir, Turkey; 3 Department of Medical Microbiology, Faculty of Medicine, Inonu University, Malatya, Turkey; 4 Department of Internal Medicine, Faculty of Veterinary Medicine, Firat University, Elazig, Turkey; 5 Department of Virology, Faculty of Veterinary Medicine, Ataturk University, Erzurum, Turkey

**Keywords:** Feline tuberculosis, Pathological findings, *Mycobacterium bovis*, Systemic involvement

## Abstract

**Background:**

The diagnosis of previous cases of feline tuberculosis in Turkey has been made based solely on pathological changes without isolation of the causative agent. This case report details the first case of feline tuberculosis in Turkey for which the causative agent (*Mycobacterium bovis*) was confirmed with microbiological isolation, morphological evaluation, molecular (PCR) characterization and antibiotic sensitivity.

**Case presentation:**

Systemic tuberculosis was diagnosed via postmortem examination of a 5-year-old stray male cat. *Mycobacterium bovis* was isolated from the lungs, bronchial and gastrointestinal lymph nodes, kidney and liver. The isolate was defined as *M. bovis* using the Genotype MTBC assay (Hain Lifescience, Germany), which allows differentiation of species within the *Mycobacterium tuberculosis* complex with an easy-to-perform reverse hybridization assay.

Pathological changes were characterized by multifocal to coalescing granulomatous inflammation in the lungs, liver, lymph nodes and kidneys. Further pathological changes included severe, diffuse, hepatocytic atrophy, periportal fibrosis with lymphohistiocytic infiltration, multifocal lymphohistiocytic interstitial nephritis, mild focal pulmonary anthracosis and mild renal and hepatic amyloidosis. Infection by immunosuppressive viral pathogens including feline herpes virus-1, feline immunodeficiency virus and feline parvovirus virus were ruled out by polymerase chain reaction assay (PCR). The isolated mycobacteria were susceptible to isoniazid, ethambutol, rifampicin or streptomycin.

**Conclusion:**

Disseminated *M. bovis* is a rare infection in cats. Involvement of submandibular lymph nodes suggested that primary transmission might have been the oral route in the present case.

## Background

Bacteria of the *Mycobacterium* genus are nonsporulating, acid-fast and weakly gram-positive bacilli measuring 1 to 4 μm in length and 0.3–0.6 μm in width. They are straight or mildly curved rods that are most commonly associated with disseminated infections in specific hosts. The genus includes *Mycobacterium (M.) tuberculosis* complex (*M. tuberculosis, M. bovis, M. africanum, M. microti*) and the *M. avium* complex [1]. *M. bovis*, which is the causative agent of tuberculosis in domestic cattle, wild and domestic animals, and occasionally in humans, is of concern throughout the world [2, 3]. *M. tuberculosis complex* infections are characterized by triggering a granulomatous inflammatory reaction with cell-mediated delayed-type hypersensitivity response [1]. After mycobacteria enter the body through either the respiratory or alimentary tract, they are phagocytosed by macrophages. In the case of a poor cell-mediated immune response, they can persist and grow inside the cells by inhibition of the phagosome-lysosome fusion in addition to several other defense mechanisms. These defense mechanisms make mycobacteria one of the most challenging and important pathogens worldwide [2, 3].

## Case presentation

Feline tuberculosis is most commonly caused by *M. microti* and *M. bovis*, and rarely by *M. tuberculosis* [4]. Data on the presence of feline tuberculosis in Turkey in the last two decades is limited to two case reports diagnosed soley by pathological lesions with no molecular typing or microbiological isolation [5, 6]. The aim of this report was to describe pathological, microbiological and molecular findings (PCR) in a case of naturally occurring tuberculosis caused by *M. bovis* in a stray domestic cat.

An approximately, 5-year-old female, domestic short haired cat was brought to the Veterinary Teaching Hospital of Firat University in very poor condition and died before clinical examination. According to the person who brought the animal to the hospital, the cat was a stray cat she had been feeding since it was a kitten.

**Fig. 1:**
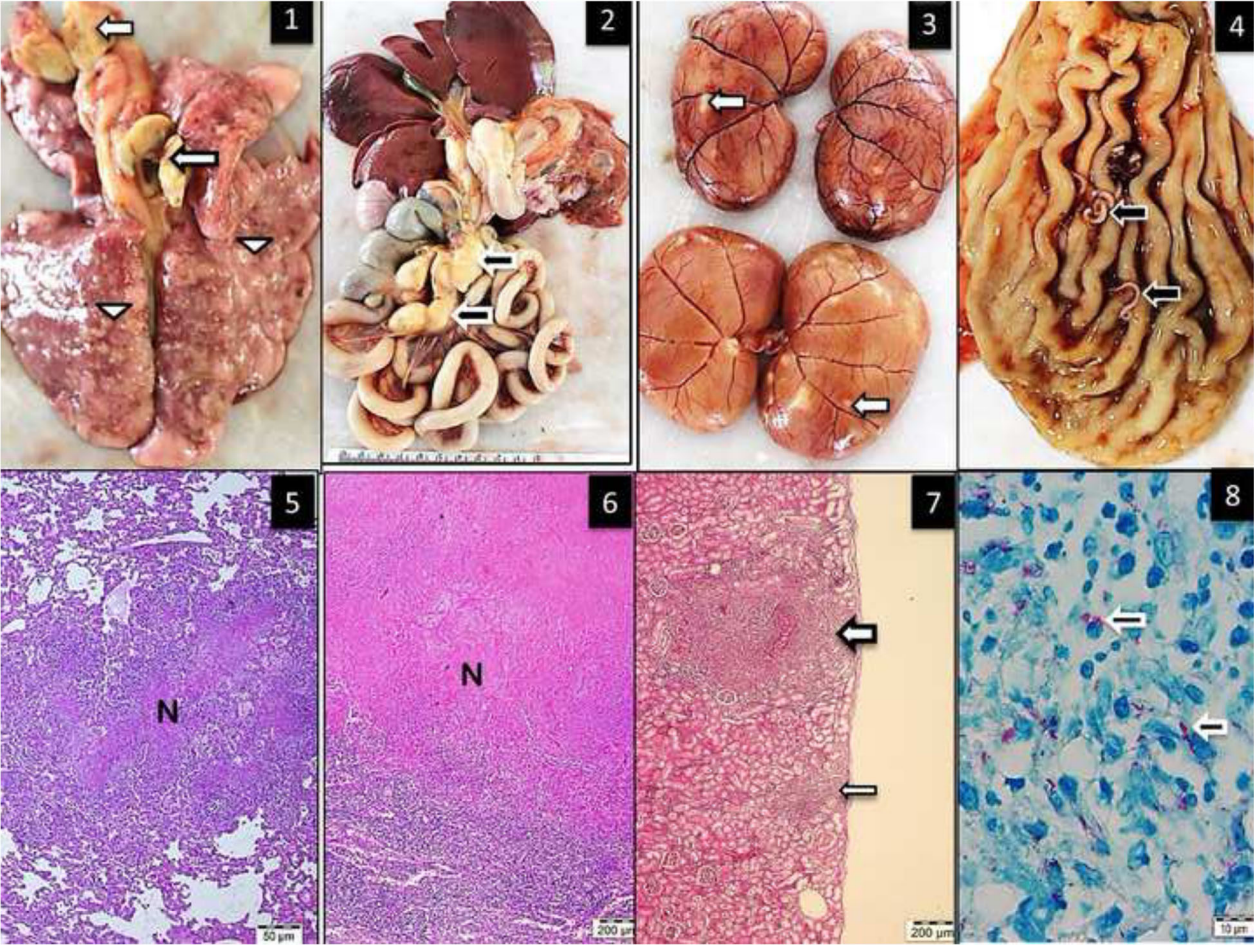
1. Multifocal granulomatous pneumonia (arrowhead) and diffuse lymphadenitis (arrow) of tracheobronchial lymph nodes. 2. Diffuse, severe lymphadenitis in mesenteric lymph nodes (arrow). 3. Multifocal granulomatous nephritis. 4. Gastric worms on the mucosal surface (arrows). 5.6.7. Granulomatous pneumonia (5) with necrosis (N), lymphadenitis (6) and necrosis and nephritis (7) with granulomas (arrows), HE. 8. Acid-fast microorganisms in the cytoplasm of epithelioid macrophages (arrows), ZN

At necropsy, the cat had extremely poor body condition and weighed 2.20 kg, and was moderately dehydrated. The most prominent gross change was multifocal white nodules of inflammation measuring 3–5 mm in diameter affecting the lungs, liver and kidneys Fig. 1 (1–3). These foci were scattered both on the surface and within the parenchyma. Submandibular, bronchial and mesenteric lymph nodes showed diffuse fibrosis and coalescing areas of caseous necrosis. Five worms (Physoleptera spp) embedded in the gastric mucosa were also present.

Samples of liver, lungs, tracheobronchial, mediastinal and mesenterial lymph nodes spleen, pancreas, brain, small and large intestines, heart, pericardium, and kidneys were fixed in 10% neutral buffered formalin, routinely processed for paraffin embedding and sectioned and stained with hematoxylin-eosin (H-E). Selected tissue sections were also stained with Ziehl-Neelsen (ZN) stain, Congo red and Masson’s trichrome.

Microscopically, multifocal granulomatous inflammation was present in the lungs, spleen, liver, kidneys and tracheobronchial and mesenteric lymph nodes Fig. 1 (6–8). Typically, most granulomas were composed of a necrotic core surrounded by epithelioid macrophages, lymphocytes, plasma cells and multinucleated giant cells. Acid-fast microorganisms were present both within the cytoplasm of macrophages, Langhans type giant cells and extracellularly within the granulomas found in the lungs, liver, kidney and lymph nodes.

Pulmonary changes included mild and multifocally distributed black pigment within macrophages (anthracosis), focally extensive intra-alveolar edema, extensive necrotizing bronchitis and diffuse, moderate alveolar histiocytosis. The liver showed widespread, severe, diffuse atrophy of hepatocytes with binucleation. Other hepatic lesions included bile duct epithelial hyperplasia, periportal fibrosis, and interstitial lymphohistiocytic infiltration. There was focally extensive amyloid deposition in the hepatic spaces of Disse and renal glomerular tufts and medullary interstitium, confirmed by Congo red staining. There were also multifocal lymphohistiocytic infiltrates in the renal interstitium. Additionally, there was a multifocal mixed inflammatory infiltrate containing eosinophils in the gastric mucosa. The brain and eyes were free of lesions.

Tissue samples from liver, bronchial lymph nodes, lung and kidney were minced into small pieces by a tissue crusher (TissueLyser LT- QIAGEN-Germany). Firstly tissue homogenates were digested and decontaminated as previously described by the N-acetyl-L-cysteine-sodium hydroxide (NALC-NaOH) method [7]. After centrifugation, a portion of sediment was used for preparation of direct smear for ZN staining, then it was examined under 100X magnification and evaluated according to International Union Against Tuberculosis and Lung Disease criteria. All the specimens were graded as 3+ infected. The other portion of sediment was directly inoculated onto both Lowenstein-Jensen (LJ) medium slants and the VersaTrek myco bottles (Thermo Fisher Scientific, Canada) for automated culture according to the manufacturer’s specifications. The LJ medium cultures were incubated at 37 °C in the incubator and examined weekly for 5 weeks. Mycobacterial growth was assessed by colony formation on solid media or positive growth signal by the VersaTrek system and confirmed by ZN staining, GenoQUICK-MTB (HAIN, Germany) and immunochromatographic-based card test (SD Rapid test, Standard Diagnostics INC, India). All isolates were identified as *M. bovis*.

Drug susceptibility testing for first-line antituberculosis drugs was performed with the VersaTrek automated culture system according to the manufacturer’s instructions (Thermo Fisher Scientific, Canada). *M. bovis is* known to be naturally resistant to pyrazinamide but is generally susceptible to other antituberculosis agents [8]. Our isolate was susceptible to isoniazid, ethambutol, rifampicin and streptomycin, which are all first-line antituberculosis drugs [9].

For virus detection, 10 μm thick sections of paraffin-embedded spleen, lymph nodes, brain, kidney and liver of the cat were deparaffinized as previously described [10]. GF-1 viral nucleic acid kit (Vivantis, Malaysia) was used for nucleic acid extraction according to the manufacturer’s recommendations. All the extracts were kept at − 20 °C until tested. Oligonucleotide primer pairs were used for the detection of Feline Herpes Virus-1 (FHV) [11], Feline Immunodeficiency Virus (FIV) [12] and Feline Parvovirus (FPV) [13]. Primers, annealing temperatures and other amplification conditions were optimized for each primer pair.

Primers used for feline herpes virus (FHV forward, TGTCCGCATTTACATAGATGG 3 and FHV reverse, GGGGTGTTCCTCACATACAA), feline immunodeficiency virus (FIV: round 1: VE-1S: GAG TAG ATA CWT GGT TRC AAG, VE-1R: CAT CCT AAT TCT TGC ATA GC and 2nd round VE-2R: ACC ATT CCW ATA GCA GTR GC, VE-2S: CAA AAT GTG GAT GGT GGA) and feline parvovirus (FPV: Hfor –CAGGTGATGAATTTGCTACA Hrev–CATTTGGATAAACTGGTGGT).

### Optimization

PCR assays for virus detection consisted of 35 cycles of denaturation at in 94 °C for 5 min, annealing at 55 °C (FHV) or at 50 °C (FIV and FPV) for 1 min. and elongation at 72 °C.

### PCR mix components

0.5 μl of Taq DNA polymerase (5 u/μl), 3 μl of 10 X Taq buffer, 2.4 μl of MgCl_2_ (25 mM), 0.5 μl of 10 mM dNTP mix, primer-forward), 19.6 μl of deionized water and 3 μl of template DNA in total reaction volume of 30 μl. Nucleic acids of these viruses were not detected by PCR

## Discussion

Disseminated *M. bovis* is a rare infection in cats [3, 4] that typically results from ingestion of infected meat, unpasteurized milk, wild rodents or contact with contaminated material [3, 14]. It would be speculative to address the source of the infection in the present case. However, outdoor cats are reportedly higher risk for *M. bovis* infection due to small rodent predation [14]. Further, the involvement of the submandibular lymph nodes suggested the infection might have been via the oral route [4, 13]. We do not have any knowledge about the feeding of the cat except for house leftovers given. Pathogen to pathogen interactions were considered in the present report assuming the cat more susceptible to *M. bovis* infection or infection dissemination if they are immunosuppressed by FHV, FIV or FPV. However, no viral immunosupression could be detected in the present report. A large proportion (31%) of 205 lions with bovine tuberculosis was co-infected with FIV, however, no synergistic relation was present as in AIDS and bovine tuberculosis in humans [15].

Concurrent occurence of amyloidosis and anthrocosis is the classical findings for the tuberculosis in any species. Cats are not generally considered to be an important host in the epidemiology of tuberculosis; however, mycobacterial culture results in cats showed (33%) of the feline submissions were positive for *M. bovis* [14]. There is no standard therapy in feline tuberculosis; however, when treatment is pursued in small animals, it should consist of a triple combination of rifampicin, macrolide and fluoroquinolone antibiotics [16].

## Conclusion

This is the first reported case in Turkey of a cat infected with *M. bovis* that has been confirmed by molecular typing of isolates recovered from its tissues with no immunosuppression by feline viruses that might make the cat more susceptible to infection.

## Abbreviations

ARB: Acid resistant bacteria
FHV: Feline herpes virus-1
FIV: Feline immunodeficiency virus
FPV: Feline parvovirus
HE: Hematoxylin and eosin staining method
PCR: Polymerase chain reaction
ZN: Ziehl-Neelsen staining method
